# The effects of socioeconomic status and short stature on overweight, obesity and the risk of metabolic complications in adults

**Published:** 2013-09-30

**Authors:** Luz Stella Álvarez Castaño, Alejandro Estrada Restrepo, Juan Diego Gomez Rueda, Cristina Carreño Aguirre, Lorena Patricia Mancilla López

**Affiliations:** 1 School of Nutrition and Dietetics at the University of Antioquia. Medellin Colombia. Research Group: social determinants of health and nutrition status.; 2 School of Nutrition and Dietetics at the University of Antioquia. Medellin Colombia. Research Group: Demography and health.

**Keywords:** Social inequality, nutritional status, obesity, overweight, heath inequalities, social class

## Abstract

**Objective:**

to observe the relationship between socioeconomic status, height and nutritional problems related to obesity, overweight and risk of metabolic complications in men and women of Medellin (Colombia).

**Methods:**

cross-sectional study with a sample of 5556 adults between 18 and 69 years of age. We assessed weight, height and waist circumference. Socioeconomic variables were evaluated by family income, socioeconomic stratum and academic level achieved.

**Results:**

we found that in men and women the height reached in adulthood is associated with socioeconomic conditions as measured by the socioeconomic strata and family income. In women, height, age, and socioeconomic strata are associated with obesity, overweight and risk of obesity, and risk of metabolic complications.

**Conclusion:**

These results are not only from individual unhealthy habits, such as eating patterns based on high density foods combined with low energy expenditure, but also from the cumulative effect of food deprivation throughout life. Therefore, policies intended to prevent them should take a preventive approach that begins before birth and continues during childhood and adulthood.

## Abstract (es)

**Objetivo:**

Observar las relaciones entre estatus socioeconómico, estatura y problemas nutricionales de sobrepeso, obesidad, riesgo de complicaciones metabólicas en hombres y mujeres de Medellín.

**Métodos:**

estudio transversal, descriptivo con una muestra de 5556 adultos con edades entre 18 y 69 años. Se evaluaron peso, estatura y perímetro de cintura. Como variables socioeconómicas se evaluaron los ingresos familiares, el estrato socioeconómico y el nivel educativo.

**Resultados:**

Se encontró que en hombres y mujeres la estatura alcanzada está asociada con las condiciones socioeconómicas medidas por estrato e ingresos familiares. En las mujeres la estatura alcanzada, la edad y el estrato están asociados con la obesidad, el sobrepeso y los riesgos de complicaciones metabólicas.

**Conclusión:**

la obesidad, el sobrepeso y el riesgo de complicaciones metabólicas en la adultez son el resultado no solo de hábitos individuales no saludables como patrón alimentario basado en alimentos altamente energéticos, sumado a bajo gasto calórico, sino también a una acumulación de privaciones alimentarias a lo largo de la vida y deben ser intervenidas desde la etapa C, la infancia y en la adultez.

## Introduction

Obesity and overweight are defined as the abnormal or excessive accumulation of fat with the potential for adverse health effects^1^. In adults, both conditions increase the risk of cardiovascular disease, diabetes and several types of cancer, as well as non-fatal conditions such as arthritis. In addition, excess abdominal fat, as measured by waist circumference, is associated with an increased risk of cardiovascular disease and other pathologies^2^. Overweight and obesity are the world´s fifth leading risk factors for death. Each year at least 2.8 million adults die as a result of these conditions^1^. 

Obesity, previously considered an exclusive problem of high-income countries, is increasing in prevalence in low and middle-income countries, especially in the Americas and Africa. In 2008, according to the World Health Organization, one billion five hundred million adults ≥20 years of age were overweight. Of this number, more than 200 million men and nearly 300 million women were obese^1^. By 2015, it is predicted that about 2.3 million adults will be overweight and more than 700 million people will be classified as obese^3^.

According to data from the latest National Survey of Nutritional Status (ENSIN 2010), in Colombia among adults between 18 to 64 years old, 34.6% were overweight and 16.5% were obese. Obesity is more prevalent in women (20.1%) than in men (11.5%). Nationally, 62.0% of women and 39.8% of men had abdominal obesity as measured by waist circumference, with the highest rates observed in people over 50 years old^4^.

In the last two decades, several studies and reviews have tried to determine the relationship between overweight or obesity and socioeconomic conditions in countries with differing levels of development. In some developed American and European countries, obesity is inversely related to socioeconomic status in women, while this relation is less consistent in men^5^. In developing countries, the results show both positive and negative associations between obesity and socioeconomic status. For example, McLaren found that low socioeconomic status is associated with increased obesity in women from more developed countries when education and occupation are used as indicators; however, in under- and mid-developed countries, there is a positive association between socioeconomic status and obesity in women when income and property indicators are used as parameters. Thus, these patterns may differ depending on the level of development of the country and on the socioeconomic status indicator used^6^. 

Similarly, short stature is an indicator of inadequate nutrition and low socioeconomic status during childhood^7^. Some studies have associated malnutrition at an early age with obesity^8^ ^,^ ^9^, and research conducted on adults in Europe and America has found positive associations between short stature, the prevalence of obesity^9^, morbid obesity, cardiovascular risk and diseases related to glucose metabolism. 

According to the thrifty phenotype hypothesis, nutritional deprivation in early life leads to adaptive mechanisms that could result in increased susceptibility to obesity in adulthood. In addition, chronic malnutrition in children results in a reduction in linear bone growth and therefore an increased risk of obesity^10^ ^,^ ^11^.

This study explores the relationship between socioeconomic status, height and being overweight or obese and the risk of metabolic complications in men and women in Medellín, Colombia. The purpose of the study is to analyze how economic and social factors throughout life are reflected in the health of individuals as adults, especially in women. We hypothesized that delayed growth due to unfavorable conditions during childhood and throughout life predispose women to being overweight and obese in adulthood.

## Material and Methods

Data were drawn from the "Food and Nutritional Profile of Medellín 2010" study, conducted by the local government of Medellín (Alcaldía Municipal). The study was descriptive and cross-sectional: the sampling process was random, multi-staged and stratified. The study was carried out during the first half of 2010. The total sample consisted of 2,719 households and 5,556 adults between the ages of 18 to 69 years (3,431 women, and 2,125 men were involved)^12^.

A household was understood as a person or group of persons living in a dwelling either full-time or part-time. The fundamental requirement was to share food and to recognize the person exercising authority as the head of household. Persons who shared the same place but did not meet these requirements were considered as another household. 

Each household was visited and a survey was administered to capture individual and socio-economic status data. At that time, the nutritional status variables and participants' age data were gathered for all family members present; a follow-up visit was scheduled to gather data from absent household members. After receiving three weeks of training, the information was gathered by students in the eighth semester of the School of Nutrition and Dietetics. The full methodology is described elsewhere^12^.

### Socioeconomic-status variables

Social stratum, educational level, and family income were established as socioeconomic-status variables. The social stratum is a classification system used in Colombia that divides households into six class levels defined by housing and environmental characteristics and sorted in ascending order. Thus, households and neighborhoods in more economically precarious conditions are classified as stratum one, and the homes and neighborhoods with the most affluent conditions are classified as stratum six. This information was confirmed by means of examining a current utility bill which was requested from each respondent^12^. For this study, the strata were classified into three groups: low (strata one and two), medium (strata three and four) and high (strata five and six). 

Educational levels were grouped into three categories: none/primary (complete or incomplete), secondary (complete or incomplete) and higher, which includes technological and university levels (complete or incomplete). This variable was set according to the highest completed grade. Due to the low frequency of the category ¨No education¨, or illiteracy, and considering that it behaves similarly to the primary educational category, we decided to merge the two levels under the label none/primary.

Household incomes were set according to whether they were sufficient to ensure food security; the price of basic food staples in Medellin was calculated for 2010 at 570,000 Colombian pesos ($316 USD). According to the Economic Commission for Latin America and The Caribbean (CEPAL), a comparative equivalence of household expenditures in different countries in Latin America by the law of Engel^13^, Colombian families spent between 30-35% of their income on food, hence a monthly income of at least 1,400,000 Colombian pesos was required to ensure food security. Thus, this variable was grouped into two categories: ≤$1,400,000 (<$796 USD) and >$1,400,000 (>$796 USD).

### Nutritional status variables

Measures of weight, height and waist circumference were taken as nutritional status variables. For the measurement of body weight, a digital brand (TANITA) was used, with a capacity of 150 kg and 0.1 kg of sensibility. Individuals were weighed without shoes, but with light clothing and minimal accessories. Height was measured with a portable meter (SECA) with a length of 200 cm and sensibility of 0.1 cm. The waist circumference was measured with a non-retractile metric tape (MABIS), with a length of 150 cm and a sensitivity of 0.1 cm. This measurement was taken by marking the last rib and the top edge of the iliac crest on both sides, and the tape was situated at the midpoint of these two points without squeezing the tissue of the skin. The reading of the measurement was conducted in an expiratory state. The measuring equipment was checked daily to maintain accuracy. In the case of the digital scale, a pattern of 20 kg was used to check its operation. In addition, interviewers were trained to collect data on body weight, length/height and waist circumference, according to standardized procedures of the World Health Organization^14^.

### Data analysis

Height was divided into quartiles by sex, according to data drawn from this study. For men, the following groups were established: ≤163.8 cm (bottom quartile), 163.9 to 168.6 cm, 168.7 to 173.0 cm and, finally, ≥173.0 cm (top quartile). For women, the categories were as follows: ≤151.1 cm (bottom quartile), 151.2 to 155.4 cm, 155.5 to 159.8 cm and ≥159.8 cm (top quartile).

Definitions for overweight or obesity were defined by calculating the body mass index (weight in kg/height in m^2)^, and the cut-off points established by the World Health Organization (WHO) were used. According to the WHO, a BMI of 25 kg/m^2^ or greater is considered overweight. A BMI of ≥30 kg/m^2^ is considered obese^1^.

The risk of metabolic complications was established based on abdominal obesity, as measured by waist circumference. Waist measurements of 80-87 cm for women and 94-101 cm for men are considered a high risk factor for metabolic complications according to the parameters set by the WHO^15^. In this study, we used the WHO parameters, and values above the defined range indicated a very high risk in both sexes.

The data were described by a relative distribution of frequencies. We used the Spearman rank correlation test to analyze the association of the values of socioeconomic status, age, educational level and risk of metabolic complications to each height quartile. Tests for association of the quartiles to income, obesity and overweight were performed using Chi-squared tests. Then, we fitted a crude logistic regression model for each sex to obesity, overweight allowed and risk of metabolic complications, with height as an independent variable. A second model adjusted height to socioeconomic status, age, sex, income and educational level. These models allowed for the calculation of the odds ratios (OR) and the 95% confidence interval (CI) was used. Additionally, regression trees were obtained with the purpose of determining the prevalence of overweight and obesity for each sex, using height, age, educational level, socioeconomic status and income as independent variables. The statistical analysis of the data was performed using SPSS(r) v18 for Windows.

### Ethical considerations

The ethics committee of the Faculty of Dentistry of the University of Antioquia endorsed protocols and tests needed to perform the study "Food and nutritional profile of households in Medellín 2010." The study fulfilled the ethical principles contained in the Helsinki Declaration. People who decided to participate read and signed the informed consent agreement. 

## Results

### The relationship between height and socioeconomic factors.

In men and women, the youngest (18-30 years), as well as those of highest socioeconomic status and those whose families had incomes above $1,400,000 ($796 USD), were more likely to be in the upper quartile or the top two quartiles of height, i.e., they are the tallest (Table 1). No definite pattern was found in relation to education. 

**Table 1:**
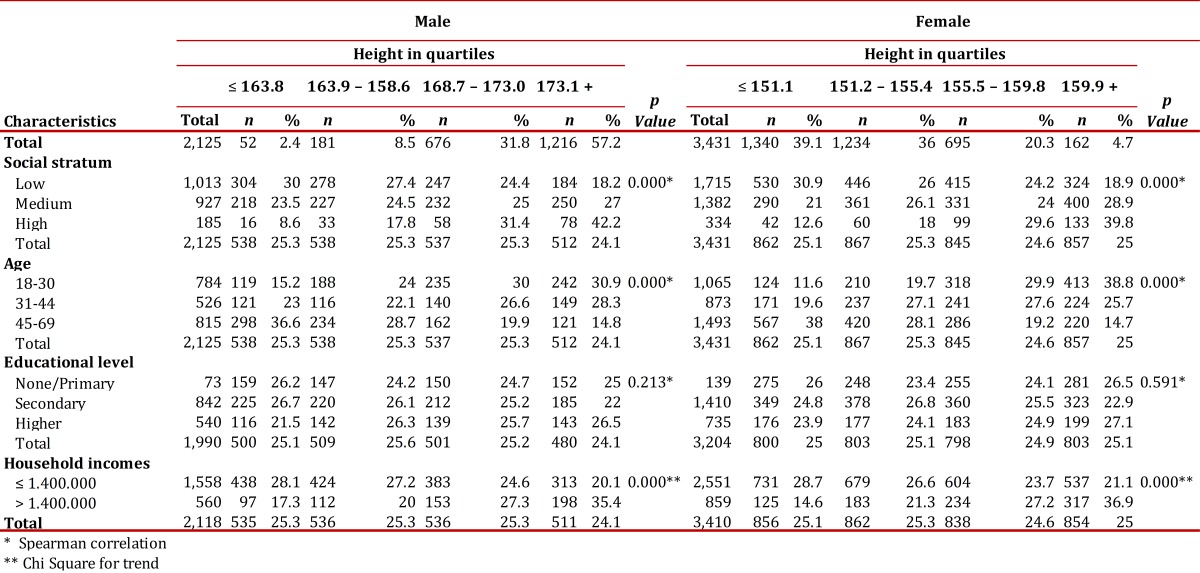
Relationship between height and socioeconomic factors

On average, 37.1% of men were overweight, 11.4% were obese and 27.7% were at high or very high risk of metabolic complications. The prevalence of overweight (39.8%) and obesity (15.1%) was higher in men in the lowest height quartile than in those in the upper quartiles, although the association was significant only for obesity (*p*= 0.004). The prevalence of obesity in men in the lowest height quartile was 15.1%, versus 9.8% in the top quartile. The risk of metabolic complications (RMC), by contrast, was higher in taller men (29.7%) compared to those in the lowest quartile (27.9%); however, the association was not significant (*p*= 0.567) (Table 2).

On average, 33.1% of women were overweight, 19.1% were obese, and 55.5% were at high or very high risk of metabolic complications, with the highest prevalence occurring in those of shorter stature. The prevalence of overweight was 27.1% in the top quartile versus 38.7% in the bottom quartile. The prevalence of obesity was 9.5% in the top quartile versus 29.1% in the bottom quartile; the RMCs were 45.0% to 64.8%, respectively. That is, in women, height is significantly and negatively correlated with overweight, obesity and RMC (*p *<0.001) (Table 2).

**Table 2:**
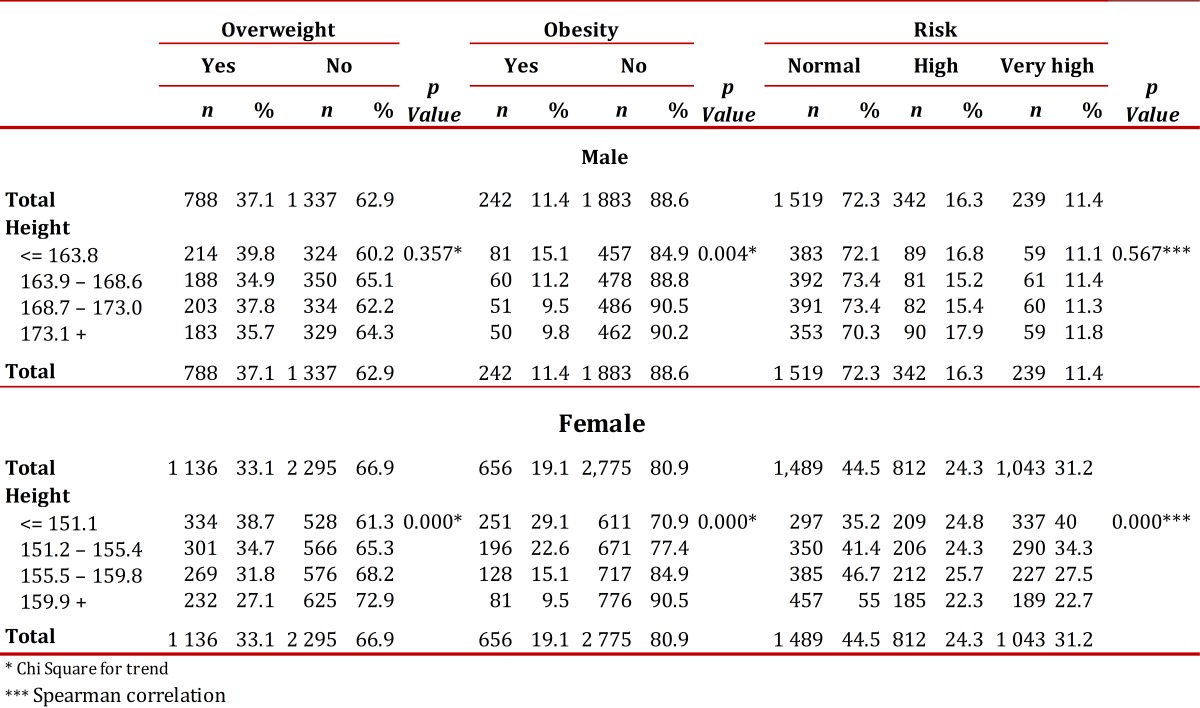
Relationship between height and nutritional status

### Relationship between height, socioeconomic factors and nutritional status

We analyzed the prevalence of overweight, obesity and RMC according to height and socioeconomic factors (education, income and socioeconomic stratum). The prevalence of obesity was highest in women in the lowest socioeconomic stratum (21.6% vs. 18.6% and 8.4%, *p* <0.001), as well as in those with family incomes under $1,400,000 ($796 USD) (*p* <0.001) relative to those with higher incomes (21.4% and 12.8%); however, no differences were observed with respect to educational level (*p*= 0.4766). A similar pattern was found in the prevalence of high RMC by family income (*p* <0.001), socioeconomic stratum (*p* <0.001) and educational level (*p* = 0.6368). 

Furthermore, for the three socioeconomic variables analyzed, the prevalence of obesity and RMC in women were highest in those of shorter stature and were negatively correlated with height (*p* <0.001), except in the highest socioeconomic stratum (*p*= 0.439 and *p*= 0.849, respectively). In men, it was not possible to establish a pattern in the association between educational level (*p* >0.05) and height, but the prevalence of obesity was negatively correlated with increasing height in men of low socioeconomic status and at the lower income levels (*p* <0.05). Similarly, the prevalence of RMC was lowest in the upper stratum and at incomes greater than $1,400,000 (*p* <0.01). In men, significant differences in the prevalence of RMC were found only by socioeconomic status (low= 25.3%, mean= 28.8%, high= 35.4%, *p*= 0.014). 

The relationship between height and nutritional status was also analyzed using gender-segregated logistic regressions in which obesity, overweight and RMC were the dependent variables in a model adjusted for socioeconomic status, age, educational level and income level. In both cases, the reference group was the highest quartile for height. In men, the probability of obesity was slightly higher for those of shorter stature (OR= 1.2), although the relationship was not significant. By contrast, this group had a significantly lower probability of RMC (OR= 0.5). Women in the lower two quartiles of stature were more likely to be obese (OR= 2.2 and OR = 1.9) (*p*= 0.00). In the logistic model adjusted for women, the ORs of the lower height quartiles for overweight and RMC were not significantly different from those of the upper quartile (Table 3).

**Table 3.**
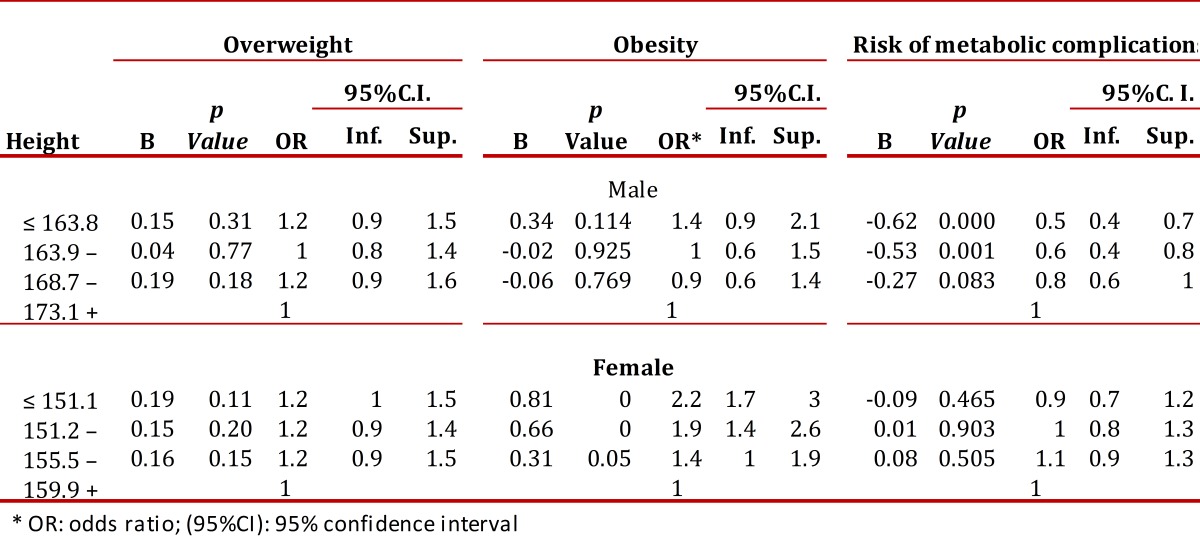
Logistic regression with model adjusted for socioeconomic status, age, educational level and income for men and women.

### Classification trees for overweight

In women, as noted above, the primary node indicated that 33.1% were overweight. The first predictor that emerged was height: of 334 women in the lowest height quartile, 38.7% were overweight, while among women in the top quartile, 27.1% were overweight. Of the women in the middle quartiles, 33.3% were overweight. 

The prevalence of overweight was 42.9% among women in the lowest quartile for height that had no or only primary-level education. This figure was slightly reduced (33.1%) among women in the same height range who had completed secondary education. Only 15.7% of the tallest women between 18 and 30 were overweight.

Among the men, 37.1% were overweight. The first predictor that emerged was height. Of 214 men in the lowest quartile for height, 39.8% were overweight. Of the men with heights above the lower quartile, 36.2% were overweight. 

The prevalence of overweight was 61.3% for men with heights above the bottom quartile who were older than 30 years and belonged to the upper social stratum. At the same time, the prevalence of overweight was 12.2% for men between 18 and 30 years old and ≥173.1 cm in height that were in the bottom socioeconomic stratum.

### Classification trees for obesity

**Figure 1:**
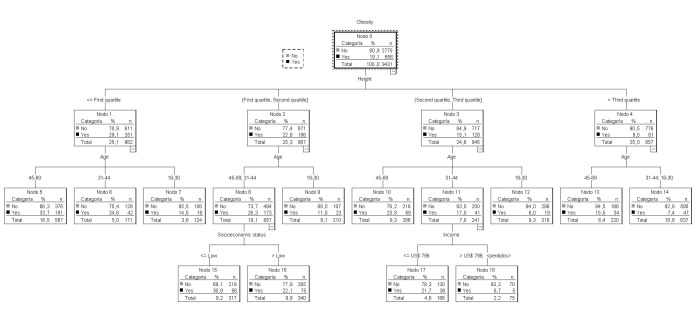
Regression tree for obesity in women

**Figure 2:**
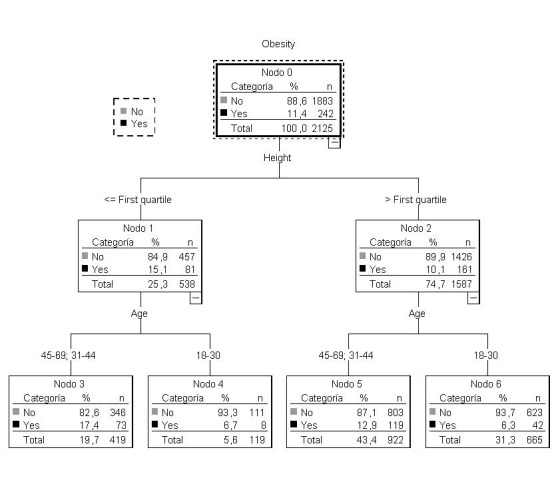
Regression tree to obesity in men

In women, the obesity rate increased from 19.1% (the overall rate) to 29.1% in the bottom quartile for height (<155.1 cm). For women between 45 and 69 years old, this percentage increased to 33.7%. On the other hand, the obesity rate increased to 30.9% in women in the second quartile for height who were >30 years old and who belonged to the lowest socioeconomic stratum. The obesity rate dropped to 6.7% for women in the third quartile for height who were aged 31 to 44 years and who earned more than $1,400,000 ($796 USD) (Fig 1). The lowest obesity rates were found in younger women (18 to 30 years) in all height quartiles. On the other hand, the prevalence of obesity increased from 11.4% (the prevalence overall) to 15.1% for men in the lowest quartile for height. Additionally, in men >30 years old, this percentage increased to 17.4%. By contrast, the prevalence of obesity increased to 12.9% among men >30 years old who were above the lower quartile for height (Fig 2). The lowest obesity rates were found in the youngest men (18 to 30 years old) in all height quartiles.

## Discussion

In both men and women, the height reached in adulthood is associated with socioeconomic conditions, as measured by the socioeconomic stratum and the family income. That is, individuals who live in more economically precarious households and neighborhoods and have families that cannot ensure food security are shorter on average than those living in better conditions. 

In women, height, age and socioeconomic strata are associated with obesity, overweight and RMC. Older age, lower socioeconomic status, and shorter height are risk factors for these health problems. Among men, only height and age are associated with these diseases. 

Our results are consistent with findings from studies conducted in Brazil and Mexico, where short stature is a known risk factor for obesity and overweight among women^7^ ^,^ ^8^ ^,^ ^16-^ ^,^ ^19^. In addition, our findings are similar to those from other studies in Brazil and Chile, which reported a higher prevalence of overweight and obesity in people with shorter statures and low incomes^16^. 

The results of our study were also consistent with other studies^6^ in that socioeconomic conditions manifested themselves differently according to sex; women belonging to lower socioeconomic strata had a higher prevalence of obesity and overweight than men and, therefore, were more likely to develop adverse health effects. Related to the association between socio-economic conditions and RCM in women, our study is consistent with findings from other investigations, such as those from the CARMELA Study and ENSIN (National Survey of Food and Nutrition 2010), which found a higher prevalence of obesity and RCM among those who belonged to the poorest strata^4^ ^,^ ^20^.

The findings regarding men are also consistent with the results found in Egypt using data from the National Obesity Assessment in 2002. In that study it was shown that shorter men were more likely to be obese, and that morbid obesity was twice as prevalent in shorter men as in men of average height^21^.

In Medellin, as in larger Colombian cities, obesity and overweight are a growing public health problem given its magnitude and its increasing prevalence in the general population. The processes associated with these two phenomena are social and individual in scope. Scientific literature widely recognizes a "nutritional transition" that consists of people worldwide consuming more high-caloric foods, saturated fats and sugars. This change in eating patterns accompanied by a reduction in physical activity that characterizes most occupations, as well as the increased use of mechanized transportation results in a positive energy balance at the individual level. As a consequence, an increasing prevalence of overweight and obesity and non-communicable chronic diseases occurs^22^ ^,^ ^23^. Although it is a widespread problem, it is still necessary to analyze its distribution, which reflects societal inequities. Its main victims are those people who do not have the necessary household income to ensure food security. Additionally, these individuals belong to the poorest strata of the population, indicating that they live in homes built with inferior materials and in neighborhoods with poorer urban facilities. These people, in turn, tend to be shorter as a result of poor living conditions in childhood. The lack of sufficient nutrients and/or recurrent infectious diseases in childhood likely prevented these adults from reaching the height they would have attained under favorable environmental conditions.

Thus, overweight and obesity in adulthood reflect the accumulation of disadvantages throughout life^24^ ^,^ ^25^. The foundation of the problems encountered in adulthood lies not only in the unhealthy individual habits already mentioned, such as excessive intake of calories and low physical activity levels, but also in poor living conditions that contribute to poor health at each stage of development. For this reason, policies intended to prevent chronic problems associated with obesity and overweight, such as diabetes, hypertension and metabolic syndrome, should take a preventive approach, starting before birth and continuing through childhood. Consequently, to encourage individuals to eat healthy foods and to be physically active, such laws should guarantee the right to sufficient and nutritionally balanced food. Additionally, these policies should consider gender, given the evidence that socioeconomic conditions disproportionately affect the nutritional status of women. 
